# Supplementary material for: Reexamination of N-terminal domains of syntaxin-1 in vesicle fusion from central murine synapses
Source: eLife. 2021 Aug 24;10:e69498. doi: 10.7554/eLife.69498 (PMC8416022; doi:10.7554/eLife.69498)
Supplement: Transparent reporting form [file elife-69498-transrepform1.docx]

***eLife’s* transparent reporting form**

We encourage authors to provide detailed information *within their submission* to facilitate the interpretation and replication of experiments. Authors can upload supporting documentation to indicate the use of appropriate reporting guidelines for health-related research (see [EQUATOR Network](http://www.equator-network.org/%20)), life science research (see the [BioSharing Information Resource](https://biosharing.org/)), or the [ARRIVE guidelines](http://www.plosbiology.org/article/info:doi/10.1371/journal.pbio.1000412) for reporting work involving animal research. Where applicable, authors should refer to any relevant reporting standards documents in this form.

If you have any questions, please consult our Journal Policies and/or contact us: [editorial@elifesciences.org](mailto:editorial@elifesciences.org).

**Sample-size estimation**

- You should state whether an appropriate sample size was computed when the study was being designed
- You should state the statistical method of sample size computation and any required assumptions
- If no explicit power analysis was used, you should describe how you decided what sample (replicate) size (number) to use

Please outline where this information can be found within the submission (e.g., sections or figure legends), or explain why this information doesn’t apply to your submission:

- All experimental designs conducted in this study are established and previously published (see references in material and methods section on pages 15-18). Therefore, calculation of an appropriate sample size and the design of each experiment could be performed based on previously published studies.

**Replicates**

- You should report how often each experiment was performed
- You should include a definition of biological versus technical replication
- The data obtained should be provided and sufficient information should be provided to indicate the number of independent biological and/or technical replicates
- If you encountered any outliers, you should describe how these were handled
- Criteria for exclusion/inclusion of data should be clearly stated
- High-throughput sequence data should be uploaded before submission, with a private link for reviewers provided (these are available from both GEO and ArrayExpress)

Please outline where this information can be found within the submission (e.g., sections or figure legends), or explain why this information doesn’t apply to your submission:

- Every data-set was obtained from 2-6 independent cultures. 5-15 individual cells were recorded for electrophysiological and Ca^2+^-imaging experiments, 30-80 images were analyzed for electron microscopy experiments, 7-10 images were analyzed for immunocytochemistry experiments, and 15 images from one well and two wells of high-density neuronal cultures were obtained for each culture.
- For electrophysiological experiments in general, the analysis was not performed for the recordings which showed a highly unstable steady-state current. In Ca^2+^-dose response experiments, the analysis was excluded when a neuron showed a high-degree of run-down effect of EPSC. For short-term plasticity measurements, the neurons which showed an initial EPSC below 100 pA were excluded. For mEPSC measurements, the neurons which involved a highly noisy background as revealed by recordings in NBQX were excluded. All these criteria are established prior to data acquisition.
- For EM analysis, the images in which an active zone could not be defined were excluded from analysis.

**Statistical reporting**

- Statistical analysis methods should be described and justified
- Raw data should be presented in figures whenever informative to do so (typically when N per group is less than 10)
- For each experiment, you should identify the statistical tests used, exact values of N, definitions of center, methods of multiple test correction, and dispersion and precision measures (e.g., mean, median, SD, SEM, confidence intervals; and, for the major substantive results, a measure of effect size (e.g., Pearson's r, Cohen's d)
- Report exact p-values wherever possible alongside the summary statistics and 95% confidence intervals. These should be reported for all key questions and not only when the p-value is less than 0.05.

Please outline where this information can be found within the submission (e.g., sections or figure legends), or explain why this information doesn’t apply to your submission:

- Statistical analysis and the justification of the statistical method used are described in Material and Methods section on page 18.
- All statistical information (mean, SEM, median, number of independent cultures, number of independent measurements, real p value for each test performed, and statistical test used) are included in individual source data files corresponding to each figure.

(For large datasets, or papers with a very large number of statistical tests, you may upload a single table file with tests, Ns, etc., with reference to sections in the manuscript.)

**Group allocation**

- Indicate how samples were allocated into experimental groups (in the case of clinical studies, please specify allocation to treatment method); if randomization was used, please also state if restricted randomization was applied
- Indicate if masking was used during group allocation, data collection and/or data analysis

Please outline where this information can be found within the submission (e.g., sections or figure legends), or explain why this information doesn’t apply to your submission:

- Groups were allocated due to their genetic background induced by viral transduction.
- Obtainment and analysis of electron microscopy images were performed blindly. No blinding was used for other experiments.

**Additional data files (“source data”)**

- We encourage you to upload relevant additional data files, such as numerical data that are represented as a graph in a figure, or as a summary table
- Where provided, these should be in the most useful format, and they can be uploaded as “Source data” files linked to a main figure or table
- Include model definition files including the full list of parameters used
- Include code used for data analysis (e.g., R, MatLab)
- Avoid stating that data files are “available upon request”

Please indicate the figures or tables for which source data files have been provided:

- We uploaded source data files which show summary tables of mean, SEM, median, number of independent cultures, number of independent measurements, real p value for each test performed, and statistical test used for each separate figure.
